# Genome-wide identification of GATA transcription factors in tetraploid potato and expression analysis in differently colored potato flesh

**DOI:** 10.3389/fpls.2024.1330559

**Published:** 2024-03-21

**Authors:** Xia Zhang, Rong Fan, Zhuo Yu, Xuerun Du, Xinyue Yang, Huiting Wang, Wenfeng Xu, Xiaoxia Yu

**Affiliations:** Agricultural College, Inner Mongolia Agricultural University, Hohhot, Inner Mongolia, China

**Keywords:** tetraploid potato, GATA transcription factor, flesh, anthocyanin, expression patterns, bioinformatic analysis

## Abstract

The GATA gene family belongs to a kind of transcriptional regulatory protein featuring a zinc finger motif, which is essential for plant growth and development. However, the identification of the GATA gene family in tetraploid potato is still not performed. In the present research, a total of 88 *GATA* genes in the tetraploid potato C88.v1 genome were identified by bioinformatics methods. These *StGATA* genes had an uneven distribution on 44 chromosomes, and the corresponding StGATA proteins were divided into four subfamilies (I-IV) based on phylogenetic analysis. The *cis*-elements of *StGATA* genes were identified, including multiple *cis*-elements related to light-responsive and hormone-responsive. The collinearity analysis indicates that segmental duplication is a key driving force for the expansion of GATA gene family in tetraploid potato, and that the GATA gene families of tetraploid potato and *Arabidopsis* share a closer evolutionary relationship than rice. The transcript profiling analysis showed that all 88 *StGATA* genes had tissue-specific expression, indicating that the StGATA gene family members participate in the development of multiple potato tissues. The RNA-seq analysis was also performed on the tuber flesh of two potato varieties with different color, and 18 differentially expressed *GATA* transcription factor genes were screened, of which eight genes were validated through qRT-PCR. In this study, we identified and characterized StGATA transcription factors in tetraploid potato for the first time, and screened differentially expressed genes in potato flesh with different color. It provides a theoretical basis for further understanding the StGATA gene family and its function in anthocyanin biosynthesis.

## 1 Introduction

Transcription factors (TFs), which are additionally referred to as trans-acting factors, are a class of proteins that can bind to *cis*-acting elements within target gene promoters. The TFs can activate or repress the expression of specific genes to regulate the transcription of downstream target genes in plants growth, development, and response to external stimuli (Franco-Zorrilla et al., 2014). Plants have diverse and complex TFs. Depending on the specific DNA sequences bound by TFs, approximately 60 TFs families with different function have been identified in higher plants (Feng et al., 2020), including MYB (Dubos et al., 2010), bHLH (Heim et al., 2003), WRKY (Rushton et al., 2010; Jiang et al., 2017), GRAS (Lee et al., 2008), and GATA families (Lowry and Atchley, 2000; Reyes et al., 2004).

GATA TFs are a kind of transcriptional regulatory proteins in eukaryotes and contain a type-IV zinc finger motif, which is highly conserved. They can specifically bind to (A/T)-GATA-(A/G) sequences in target gene promoters, which regulate downstream gene transcription levels (Lowry and Atchley, 2000). The (T/A)GATA(A/G) sequence motif was first discovered by Evans et al. (1988) in the promoters of chicken globin genes and six GATA TFs (GATA1 to GATA6) were subsequently identified to bind the motif, forming the GATA gene family. Members of the GATA family are generally composed of one or two highly conserved type IV zinc finger domains (C-X_2_-C-X_17-20_-CX_2_-C) and a downstream basic region (Lowry and Atchley, 2000). In animals, GATA TFs typically have two conserved C-X_2_-C-X_17_-C-X_2_-C zinc finger domains. However, only the C-terminal zinc finger structure plays a part in DNA binding, whereas the N-terminal zinc finger structure is in the role of regulating binding of the C-terminal zinc finger specifically to DNA or other proteins (Trainor et al., 2000). These GATA TFs can participate in growth, development, differentiation, and cell proliferation (Lowry and Atchley, 2000; Patient and McGhee, 2002). Most fungal GATA TFs include only one C-X_2_-C-X_17_-C-X_2_-C or C-X_2_-C-X_18_-C-X_2_-C domain, which has a role in multiple biological steps, including nitrogen metabolism, light response, the circadian rhythm, and siderophore biosynthesis (Teakle and Gilmartin, 1998; Scazzocchio, 2000).

In plants, the first known GATA transcription factor was identified in tobacco, which was named as NTL1 because it was the homologue of NIT2 protein discovered from *Neurospora crassa* (Daniel-Vedele and Caboche, 1993). Most plant GATA proteins feature a single C-X_2_-C-X_18_-C-X_2_-C zinc finger domain, while only several GATA proteins contain CX_2_-C-X_20_-C-X_2_-C or two zinc finger domains (Reyes et al., 2004). Studies have shown that GATA TFs played critical regulatory roles in plant growth and development. For instance, the *Arabidopsis* GATA transcription factor BME3 (At3g54810) mediates the developmental transition of seeds from dormancy to germination and positively regulates seed germination (Liu et al., 2005). The transcription factor ZIM (At4g24470) helps to regulate inflorescence and flower development (Nishii et al., 2000). The AtGATA2 (At2g45050) is an important positive regulator of photomorphogenesis and can be bound directly to the promoter elements of light-responsive and brassinosteroid (BR) genes to regulate their expression (Luo et al., 2010). With lost *GNC (At5g56860)* gene expression, chlorophyll levels are significantly reduced in mutant lines, and exogenous glucose sensitivity can vary with the change of *GNC* expression, which affects the expression of genes related to glucose signal transduction (Bi et al., 2005). The overexpression of *OsGATA8* can improve rice resistance to abiotic stress, and increase photosynthetic efficiency and biomass accumulation under both normal and saline-stress conditions (Nutan et al., 2020). The gene expression of *GATA44* and *GATA58* is significantly decreased under low-nitrogen treatment in soybean seedlings (Zhang et al., 2015). The expression levels of *FtGATA23* and *FtGATA25* genes increase significantly in Tartary buckwheat leaves under separate cold and polyethylene glycol treatments (Yao et al., 2022). Recent reports showed that GATA family members may also participate in anthocyanin biosynthesis. For example, the GATA4 protein is an important light regulatory factor and also a main regulator of the key gene for flavonoid accumulation, which is consistent with the expression level of flavonol synthase in white and pink tea flower (Zhou et al., 2020). Wang et al. (2021) identified a GATA transcription factor that negatively regulated gene related to anthocyanin accumulation by a comparative-transcriptome analysis of *Lycoris radiata* petals at different flower-development stages. Fu et al. (2021) screened 19 key TFs involved in anthocyanin accumulation from five *Camellia japonica* cultivars with different petal colors using RNA-seq, including one GATA transcription factor that positively regulated target gene expression. Fan et al. (2022) cloned candidate genes related to anthocyanin synthesis from *Camellia japonica* and identified GATA binding sites in their promoter regions. In addition, it was found that the expression level of *GATA24* was increased along with anthocyanin accumulation. The *SmGATA26* gene was identified as a hub gene in the gene regulatory network of purple peels coloration of eggplants, and the silencing of *SmGATA26* decreased anthocyanin accumulation.

Potato (*Solanum tuberosum* L.), originated in the Andean region of South America, represents the world’s fourth important food crop and is now widely cultivated in many countries and regions (Fogelman et al., 2019). As an important tuber crop, potato is characterized by drought resistance, cold tolerance, and a high yield. Potato is also a good source of nutrients, carbohydrates, and dietary fiber, with certain medical and health benefits (Camire et al., 2009). Colored potato comprises a special type of cultivated potato varieties whose tubers are rich in antioxidant anthocyanins, which has potential value in developing natural pigments, extracting antioxidants, and enhancing health benefits (Bravo et al., 2023). However, common potato cultivars are autotetraploids with rather complex genetic background, highly heterogeneous genome, and have substantial challenge in terms of genome assembly. Previous studies of potato genomics were mainly focused on diploid genome (Wang et al., 2018; Sun et al., 2019; Li et al., 2021; Huang et al., 2022). Bao et al. (2022) sequenced and completed the genome assembly of an autotetraploid cultivated potato, named as Cooperation-88 (C88),which provided valuable resources for genomics studies related to tetraploid potato.

The GATA gene family was previously identified and studied on a genome-wide basis in many different crops, such as *Arabidopsis thaliana* (Reyes et al., 2004; Bi et al., 2005), *Oryza sativa* (Reyes et al., 2004; Gupta et al., 2017), *Glycine max* (Zhang et al., 2015), *Vitis vinifera* (Zhang et al., 2018), *Brassica napus* (Zhu et al., 2020), *Triticum aestivum* (Feng et al., 2022), and *Fagopyrum tataricum* (Yao et al., 2022). However, it is not yet reported on the genome-wide identification of GATA TFs in tetraploid potato and the role in tuber anthocyanin biosynthesis.

Therefore, the aim of this study is to use bioinformatics methods to discover members of the GATA gene family at the genome-wide level in tetraploid potato, and to analyze their basic physicochemical properties, chromosomal distributions, gene structure, duplication events, phylogenetic relationships, and expression levels in different tissues. In addition, the RNA-seq was performed on two differently colored potato flesh samples, and the differentially expressed *GATA* genes were analyzed to screen the candidate genes that may support anthocyanin biosynthesis of potato tuber. It provides a theoretical basis for further investigating the function of GATA gene family members.

## 2 Materials and methods

### 2.1 Plant materials and sample preparation

**Figure 1:**
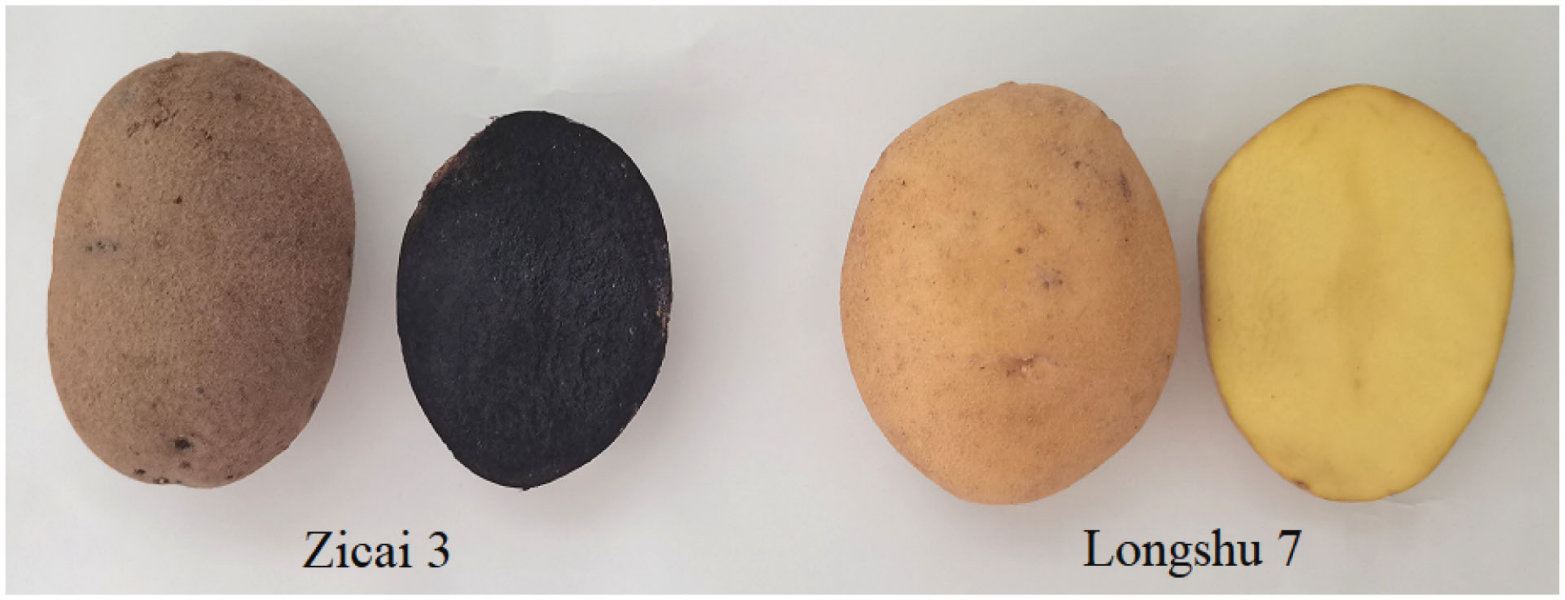
Tuber cross-sections of two potato varieties with different flesh colors.

Two tetraploid potato varieties (Zicai 3 and Longshu 7) were bred by the Agriculture College of Inner Mongolia Agricultural University and the Potato Research Institute of the Gansu Academy of Agricultural Sciences, respectively. Zicai 3 is a purple-skinned, deep-purple-fleshed variety and Longshu 7 is a yellow-skinned, yellow-fleshed variety (**Figure 1**). All experimental materials were planted at the experimental base of Agricultural College of Inner Mongolia Agricultural University (Hohhot, China; 40°46′N, 110°45′E) in May 2022. The scientific field management was conducted during the planting period to ensure normal plant growth. The samples were collected during the potato tuber-maturation stage with three replicates. After the potato tubers were cleaned with distilled water, the tuber flesh was separated. All samples were collected and immediately frozen in liquid nitrogen, and then stored at -80°C for RNA-seq and qRT-PCR analysis.

### 2.2 Identification and physicochemical characterization of GATA proteins in tetraploid potato

Based on the sequence information of the *Arabidopsis* GATA gene family (Reyes et al., 2004; Bi et al., 2005), the amino acid sequences of AtGATA members were obtained from the PlantTFDB 5.0 (http://planttfdb.gao-lab.org). The Spud DB (http://spuddb.uga.edu/c88_potato_download.shtml) was used to obtain whole-genome data of tetraploid potato. The StGATA proteins were identified using two BLAST methods. Firstly, based on the amino acid sequences of 30 known AtGATA members in *Arabidopsis*, BLAST (E-value ≤ 1e-10) searches were performed in potato protein sequences (C88.v1) to identify candidate StGATA proteins (Altschul et al., 1997). Then, the Hidden Markov Model file corresponding to the GATA protein domain (PF00032) was retrieved from the Pfam database (http://pfam.xfam.org/) as the seed sequence, and the potato protein sequences were searched using HMMER 3.3.2 software (http://hmmer.org/download.html; Finn et al., 2011) to screen for candidates containing a GATA domain. Only the longest sequence was considered as a GATA gene family member if the same gene corresponded to multiple protein sequences. Finally, duplicates were eliminated after integrating the search results from the two methods. To determine the reliability of the candidates, the integrity of their conserved domains were verified using the SMART (http://smart.embl-heidelberg.de; Letunic and Bork, 2018) and the NCBI Conserved Domain Database (https://www.ncbi.nlm.nih.gov/Structure/cdd/wrpsb.cgi; Yang et al., 2020). The protein missing a GATA domain was manually eliminated, and obtained the final StGATA proteins. Physicochemical properties such as the amino acid length, molecular weight (MW), and isoelectric point (pI) of the StGATA proteins were predicted using the online ExPASy program (http://www.expasy.org/tools/protparam/; Artimo et al., 2012). The Cell-PLoc 2.0 (http://www.csbio.sjtu.edu.cn/bioinf/Cell-PLoc-2/; Chou and Shen, 2008) was used to predict the subcellular localization of StGATA proteins.

### 2.3 Phylogenetic analysis of GATA proteins in tetraploid potato

The Cluster W (Thompson et al., 2002) was used to conduct multiple sequence alignment of reported GATA proteins of *Arabidopsis* and rice (Reyes et al., 2004; Bi et al., 2005; Gupta et al., 2017) with identified GATA proteins in potato. Then, a phylogenetic tree was constructed using the neighbor-joining method of MEGA 11 software (Hall, 2013) with 1,000 bootstrap replicates. All identified potato GATA proteins were divided into different subfamilies on the basis of the classification of the *Arabidopsi*s GATA gene family (Reyes et al., 2004).

### 2.4 Predicting conserved motifs, gene structure, and *cis*-acting elements

Conserved motifs in StGATA proteins were analyzed using the online tool MEME (http://meme-suite.org/meme/tools/meme; Bailey et al., 2009), with the maximum conserved motif search value of 10 and default parameters. The *StGATA* genes structure information was extracted using the potato genome gff3 file, and TBtools (v1.108) software (Chen et al., 2020) was used to draw a visual map of the gene structure. The 2-kb sequences upstream of the start codon of each *StGATA* gene were extracted from potato genome-wide data and used as promoter regions to identify *cis*-acting elements with the PlantCARE website (https://bioinformatics.psb.ugent.be/webtools/plantcare/html/; Lescot et al., 2002).

### 2.5 Analysis of chromosome location and gene duplication events

The chromosomal location of the *StGATA* genes were obtained from the tetraploid potato genome annotation file and were visualized using TBtools (Chen et al., 2020). To study expansion of the StGATA gene family, a phylogenetic tree was constructed based on 49 reported GATA proteins of diploid potato (Yu et al., 2022) and 88 GATA proteins in tetraploid potato. The MCScanX software (Wang et al., 2012) was used to scan the *StGATA* genes for analyzing collinearity and ascertaining the corresponding gene duplication events. Then the nonsynonymous (Ka) and synonymous (Ks) substitution rates of each duplicated gene were calculated to understand the evolutionary constraints acting on *StGATA* genes. Based on the complete genome sequences and annotation files of *Arabidopsis* and rice (downloaded from the Ensembl Plants Database; https://plants.ensembl.org/index.html), we further analyzed collinearity among GATA family members in potato, *Arabidopsis*, and rice.

### 2.6 Analysis of *GATA* gene expression levels in tetraploid potato

The RNA-seq-based expression data were downloaded from project PRJCA007997 (Bao et al., 2022) with nine different tetraploid potato tissues (including apical buds, stamens, petals, sepals, expanded leaves, stems, underground stolons, roots, and fruits) in the National Genomics Data Center (NGDC, https://bigd.big.ac.cn). The expression value (FPKM) of the *StGATA* genes was extracted, and a corresponding heatmap was generated using TBtools (Chen et al., 2020). In addition, the total RNA was extracted from different colored potato flesh samples using the RNAprep Pure Plant Kit (Tiangen Biotech, Beijing, China), and the RNA quality was examined using an Agilent 2100 Bioanalyzer (Agilent Technologies, Santa Clara, CA, USA). The qualified RNA was entrusted to sequence on an Illumina NovaSeq 6000 platform by Beijing Novozymes Technology Co., Ltd. The sequencing raw data were submitted to the NCBI (PRJNA1033052). The differentially expressed *StGATA* genes in differently colored potato flesh were analyzed based on the obtained transcriptome data (log_2_ |fold-change| >1, FDR < 0.05).

To identify association of expression levels between *StGATA* genes and key genes involved in anthocyanin biosynthesis, the correlation was investigated by Pearson’s correlation analysis and visualized using the R package “corrplot” (www.r-project.org). The statistical significance was considered as *P* value < 0.05.

### 2.7 qRT-PCR validation

We randomly selected the CDS sequences of eight *StGATA* genes that were differentially expressed in differently colored potato flesh samples and design specific primers for each gene. The qRT-PCR analysis was performed to validate their expression levels in both varieties. The total RNA of eight *StGATA* genes was reverse-transcribed into cDNA using a FastKing RT Kit (with gDNase) (Tiangen KR116, Beijing, China), and the qRT-PCR amplification was performed on QuantStudio3 & 5 PCR system (Thermo, USA).

Each qRT-PCR mixture consisted of 1.2 μL of cDNA template, 0.4 μL each of forward and reverse primer (10 μmol/L), 10 μL of MonAmp SYBR Green qPCR Mix, and nuclease-free water up to 20 μL. The thermocycling was performed with a pre-denaturation step at 95 °C for 30 s, followed by 40 cycles of denaturation at 95 °C for 10 s, annealing for 10 s (the exact annealing temperature depended on the involved primers), and extension at 72 °C for 30 s. The *U6* gene was used as a housekeeping gene (**Supplementary Table 1**). Experiments were performed with three replicates, and the relative gene expression was quantified using the 2^-ΔΔCq^ method (Livak and Schmittgen, 2001). Excel 2020 was used for variance analysis (ANOVA) of experimental data, and Origin 2022 was used to draw column graphs.

### 2.8 Predicting the tertiary structure of StGATA proteins

To further investigate the protein structure and understand its function, the SOPMA (https://npsa-prabi.ibcp.fr/cgi-bin/npsa_automat.pl?page=npsa_sopma.html) (Geourjon and Deléage, 1995) website was used to predict the protein secondary structure of differentially expressed *StGATA* genes in different colored potato flesh, and the SWISS-MODEL (https://swissmodel.expasy.org/interactive) (Waterhouse et al., 2018) online website was used to predict the protein tertiary structure.

## 3 Results

### 3.1 GATA gene family in tetraploid potato

Two BLAST methods were used to screen StGATA protein sequences, and the conserved domains were further verified by the websites SMART and NCBI-CDD. A total of 88 GATA proteins of tetraploid potato were identified, named from StGATA1 to StGATA88. Analysis of the basic physicochemical properties and subcellular localization of the StGATA proteins showed that the number of amino acids encoded by these GATA factors varied from 106 to 718, with MW ranging from 11.88 to 77.46 kD. The pI value of StGATA proteins varied from 5.33 to 9.99, and most proteins had a pI of more than 7.0, indicating that the StGATA proteins are predisposed to basic amino acid. The instability index of StGATA7 was 36.5 and that of StGATA69 was 86.49, which were the smallest and largest instability indexes of the StGATA gene family, respectively. The StGATA3, StGATA7, StGATA35, StGATA38, StGATA40, and StGATA62 were stable proteins, whereas the rest of the StGATA proteins were unstable (instability index > 40). The GRAVY value of the 88 StGATA proteins were all negative, indicating that the StGATA proteins were all hydrophilic. In addition, subcellular localization prediction showed that all StGATA family members were localized in the nucleus (**Supplementary Table 2**).

### 3.2 Phylogenetic analysis and multiple sequence alignment of StGATA proteins

**Figure 2:**
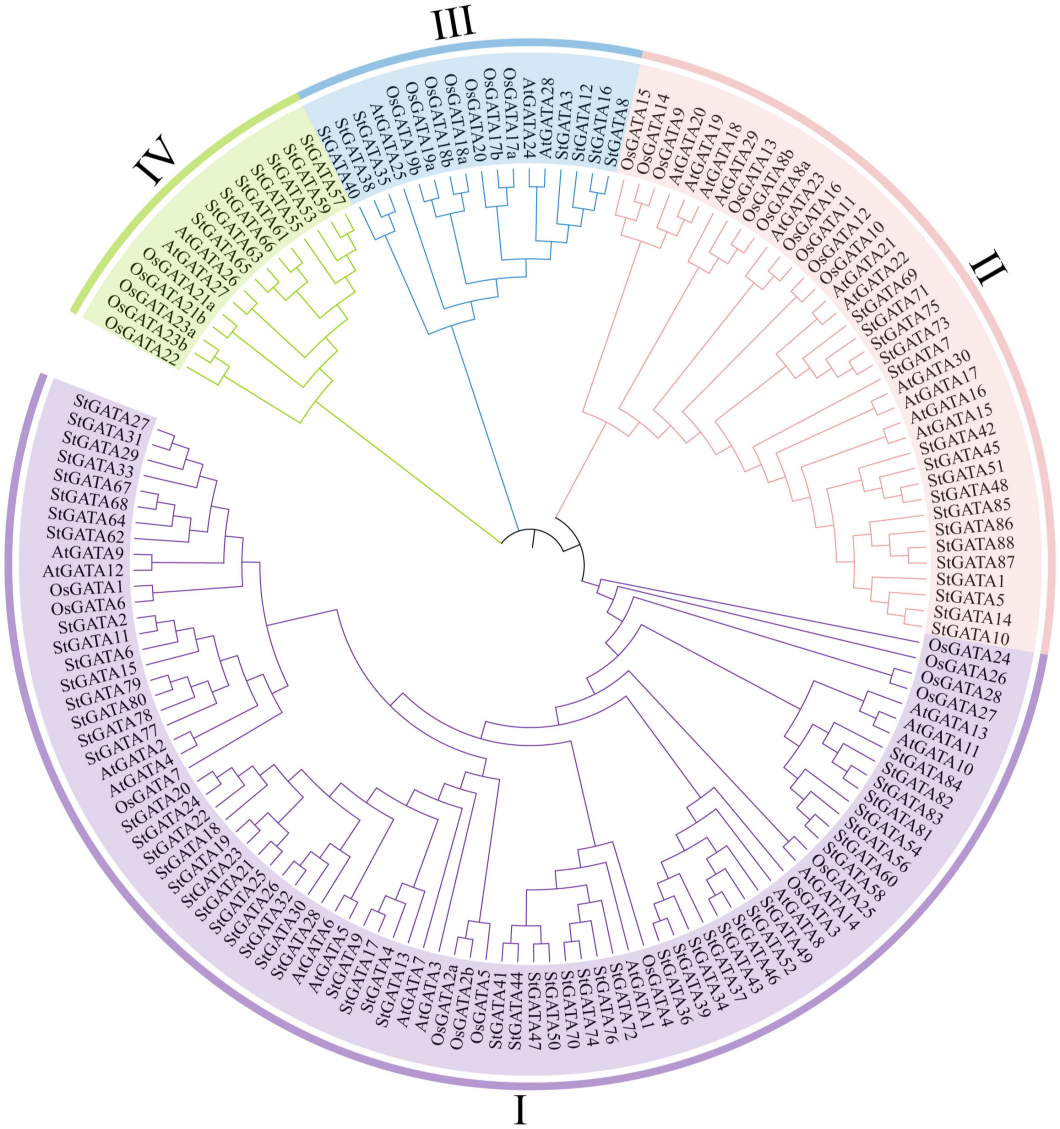
Phylogenetic tree for the GATA gene families of *Arabidopsis*, rice, and tetraploid potato. The arcs with different colors represent GATA protein subfamilies I, II, III, and IV, as indicated.

To understand the phylogenetic relationship and biological function of the StGATA family members, multiple alignment was performed on the GATA protein sequences of tetraploid potato, *Arabidopsis*, and rice, and a phylogenetic tree was constructed (**Figure 2** and **Supplementary Table 3**). The 88 StGATA gene family members were divided into four subfamilies from I to IV, according to the classification standard used for *Arabidopsis*. Among the four subfamilies, subfamily I contained the most StGATA proteins (56 StGATA proteins), accounting for 63.64% of all the identified StGATA proteins; Subfamily II contained 17 StGATA proteins; Subfamilies III and IV contained seven and eight StGATA proteins, respectively.

To further analyze the GATA domains in different subfamilies, multiple sequence alignment was performed using the amino acid sequences of the StGATA gene family. It showed that all StGATA family members just had a single GATA domain (**Supplementary Figure 1**). The StGATA domains of the other three subfamilies were all CX_2_CX_18_CX_2_C, except for subfamily III, which had a CX_2_CX_20_CX_2_C zinc finger structure. The characteristics of the GATA domains of tetraploid potato in each subfamily were consistent with those previously reported on *Arabidopsis*. In addition, the StGATA domains of all four subfamilies contained highly conserved amino acid motifs, such as TP, GP, and LCNACG, which might be related to the DNA-binding and transcriptional-activation function of GATA TFs. However, abundant variations in the GATA domains were identified between different subfamilies and within the same subfamily. For example, the amino acids following the TP conserved region showed differences on different subfamilies or the same subfamily, whereas the amino acids before the conserved LCNACG region only showed differences on different subfamilies. The variations observed at these sites are indicative of gene function differentiation among members of the StGATA gene family.

### 3.3 Gene structure and conserved motifs analysis of StGATA proteins

**Figure 3:**
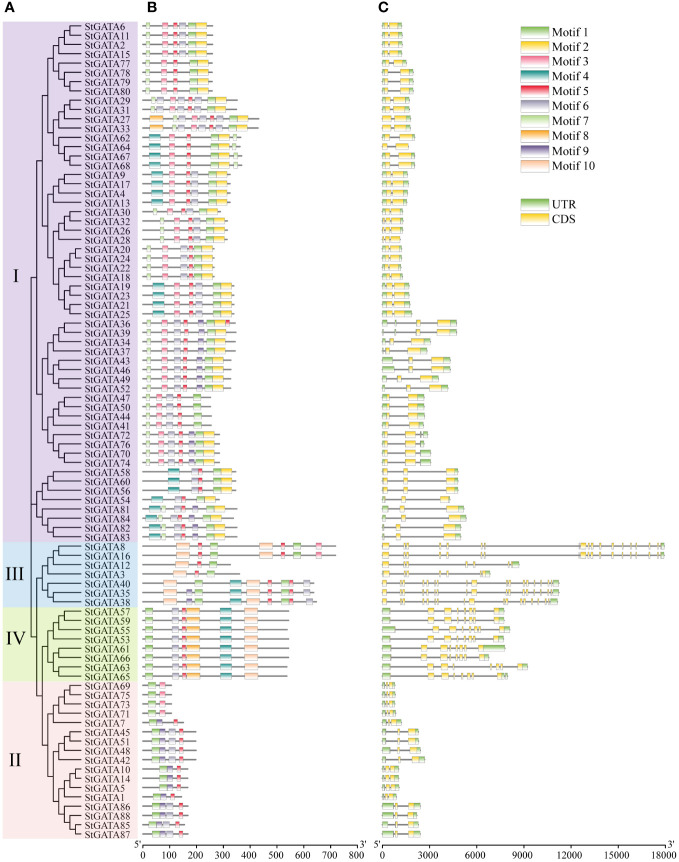
Phylogenetic tree, conserved motifs, and gene structure analysis of StGATA gene family members. **(A)** Phylogenetic tree of 88 tetraploid potato GATA proteins. **(B)** The conserved motifs of the StGATA proteins are indicated in different colors, and the associated information is indicated in the legend (top right). **(C)** The exons, introns, and untranslated regions (UTRs) are indicated by orange boxes, black lines, and green boxes, respectively.

The TBtools were applied to visualize the gene structure and construct an evolutionary tree for all 88 StGATA proteins (**Figures 3A, C**). The MEME software was applied to search for conserved motifs in the StGATA proteins (**Figure 3B**) and further define the characteristics of the StGATA gene family. A total of ten conserved motifs (motifs 1 to 10) were identified in the tetraploid potato GATA proteins (**Supplementary Table 4**), of which motif 1 was the most conserved and presented in all StGATA proteins. All other StGATA proteins contained motif 5 except for StGATA69, StGATA71, STGATA73, and STGATA75 proteins. In addition to these common motifs, each same subfamily was distinguished in terms of specific conserved motifs. For example, motifs 2 and 7 were unique to subfamily I, whereas motif 10 existed in subfamilies III and IV. In addition, all the subfamily IV members had identical conserved motifs. The gene structure analysis showed that the number of exons contained in the *StGATA* genes ranged from 1 to 19, and the number of exons varied among different subfamily genes. It was found that most *GATA* genes of subfamily III contained more than 15 exons, except for *StGATA3* and *StGATA12* with only 7 exons, the subfamily II *GATA* genes contained the fewest exons (2 exons in each *StGATA* gene), the subfamily IV *GATA* genes contained 8 exons, and subfamily I *GATA* genes contained 2 or 3 exons, respectively.

### 3.4 *Cis*-acting elements analysis of the StGATA gene family

**Figure 4:**
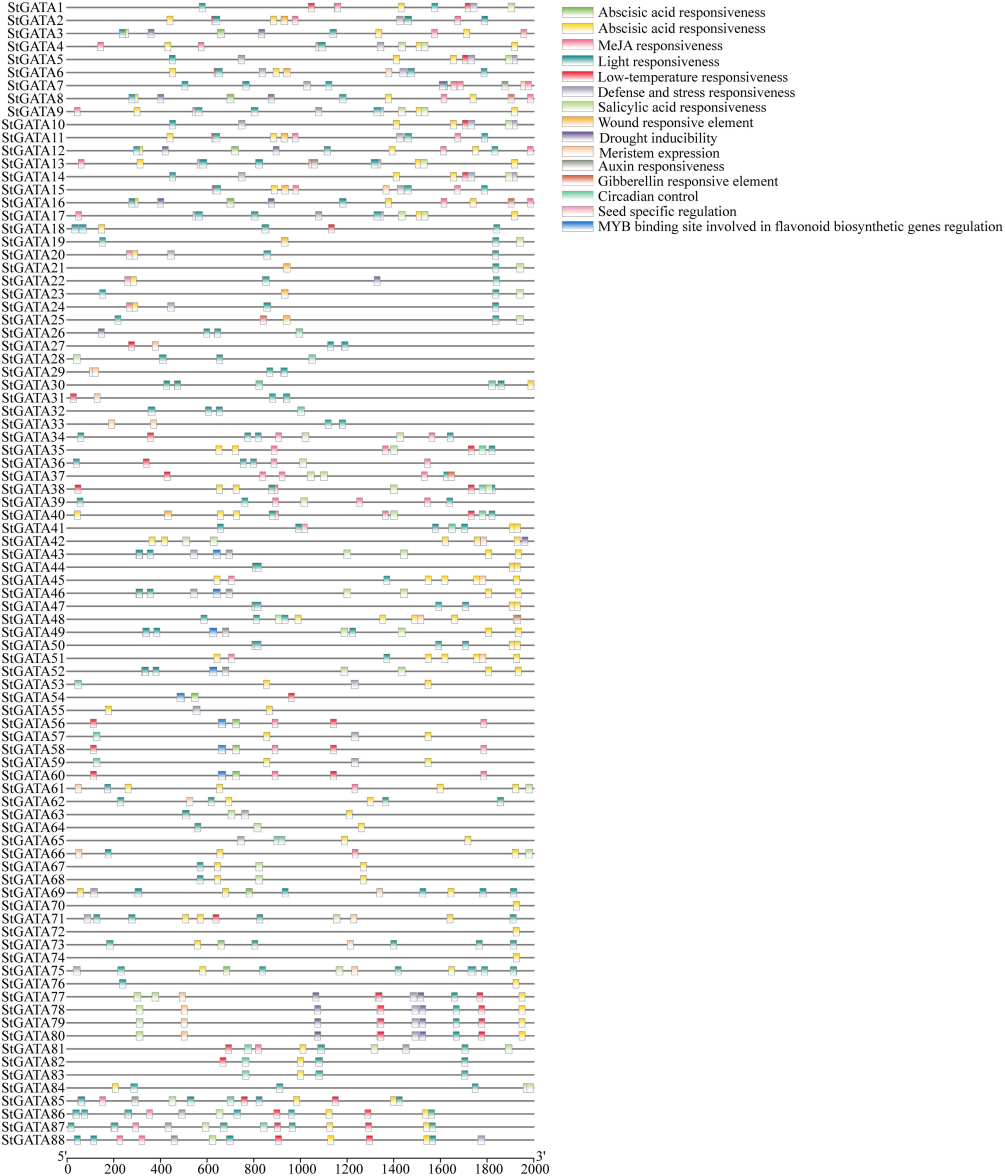
The *cis*-acting elements of the promoter sequences 2 kb upstream of all 88 *GATA* genes in tetraploid potato. The differently colored boxes represent *cis*-acting elements, as indicated in the legend (top right).

To explore the functional mechanisms of the StGATA gene family in stress response and developmental processes, the upstream 2.0 kb promoter sequences of each *StGATA* gene were extracted from the tetraploid potato genome and performed a *cis*-acting element search using PlantCARE. The *cis*-acting elements were identified among the *StGATA* genes, of which 16 different cis-acting elements were screened for comparison analysis. It showed that light-responsive elements were the most abundant and extensively existed in each *StGATA* gene promoter sequence (**Figure 4**). The hormone-responsive elements, such as the abscisic acid-responsive element (presented in 65 *StGATA* genes), were the second most abundant. Among the promoter sequences of 88 *StGATA* genes, several *cis*-elements associated with stress were identified, including those related to anaerobic induction, low temperature, and drought stress. Some *StGATA* genes have *cis*-acting elements related to defense response, mechanical damage response, meristem expression, or seed-specific function regulation. Notably, the promoters of eight *StGATA* genes (*StGATA43*, *StGATA46*, *StGATA49*, *StGATA52*, *StGATA54*, *StGATA56*, *StGATA58*, and *StGATA60*) contained MYB-binding site elements related to the regulation of flavonoid biosynthesis, implying that these genes might be directly or indirectly contributed to regulating flavonoid synthesis in tetraploid potato.

### 3.5 Chromosomal localization, gene duplication, and collinearity analysis of *StGATA* genes

**Figure 5:**
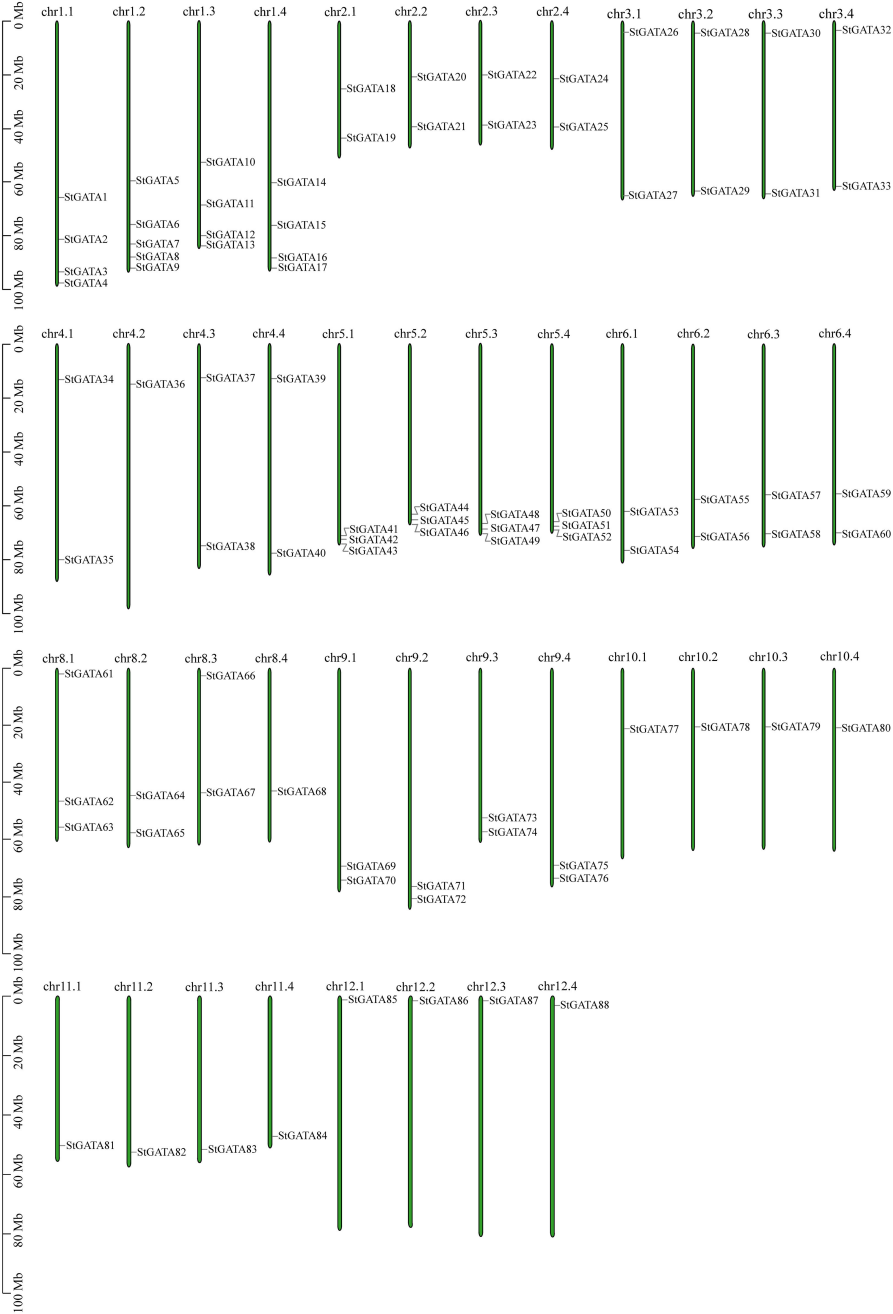
Chromosomal localization of *StGATA* genes. The green rectangular bars represent the chromosomes of tetraploid potato. The chromosome numbers and lengths (Mb) are indicated.

Based on annotation information of the tetraploid potato genome, the identified *StGATA* genes were chromosomally localized. All the 88 *StGATA* genes were unevenly distributed on the remaining 44 chromosomes except for chromosome 7 (chr7.1 to 7.4) (**Figure 5** and **Supplementary Table 6**). Of all the 44 chromosomes, the largest number of *StGATA* genes located on chr1.2 (containing five *StGATA* genes); followed by four *StGATA* genes on chr1.1, chr1.3 and chr1.4, respectively; and only one GATA gene on chr4.2, chr8.4, and chr10.1 to chr12.4, respectively. In addition, our analysis showed that the number of *StGATA* genes did not directly correlate with the chromosome length.

**Figure 6:**
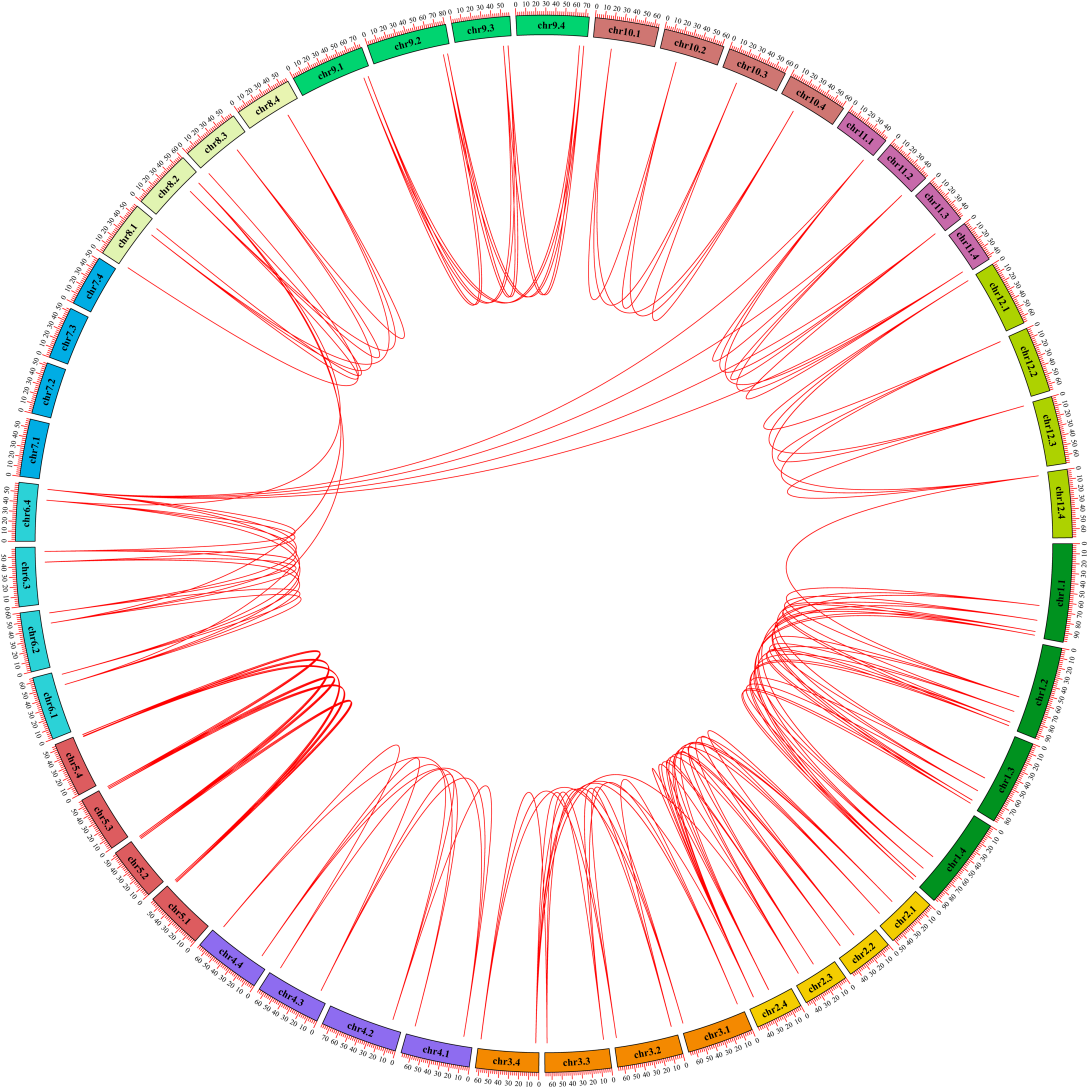
Repeated events of the StGATA gene family. The duplicated *StGATA* gene pairs are represented with red lines. The colored boxes indicate the different chromosomes, and the chromosome numbers are shown inside each chromosome.

In order to explore the gene expansion mechanism of the StGATA family during the evolutionary process, we constructed a phylogenetic tree of the GATA gene family from diploid and tetraploid potato, and analyzed the duplication events of the StGATA gene family. The number of GATA family members in diploid potato was about half of that in tetraploid potato, and the evolutionary tree was also divided the GATA family of diploid potato into four subfamilies (**Supplementary Figure 2**). Generally, the segmental duplication events exist in highly similar genes located on different chromosomes. The analysis of tetraploid potato genome showed that 98.86% (87/88) of the *StGATA* genes underwent segmental duplication but did not exist tandem duplication events (**Figure 6**). It indicates that segmental duplication events promote GATA gene family expansion. In addition, the Ka/Ks ratios of most *StGATA* homologous genes were less than 1.0 (**Supplementary Table 7**). It demonstrates that the StGATA gene family probably has undergone strong purifying selection over a long evolutionary process.

**Figure 7:**
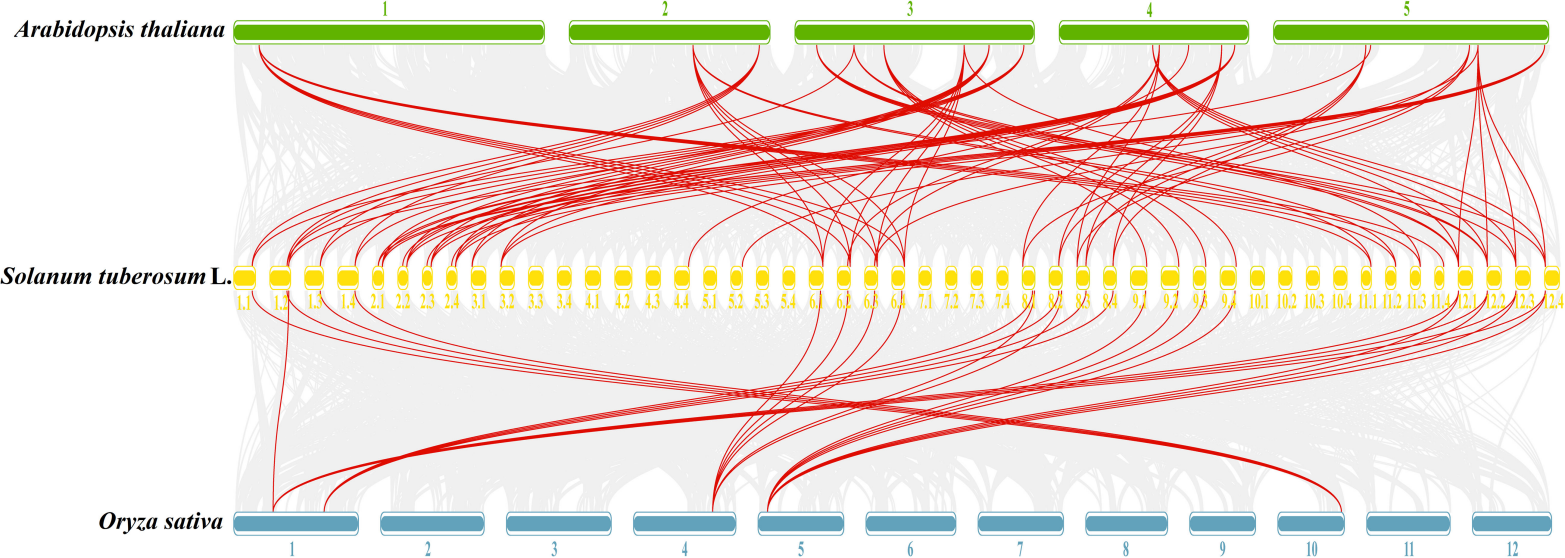
Collinearity analysis of *GATA* genes in potato, *Arabidopsis*, and rice. The background gray lines between *Solanum tuberosum* L. and other plants represent collinearity across wide regions of the genomes, whereas the red lines represent homologous relationships among *GATA* genes. The numbers represent the chromosome labels of the corresponding chromosomes.

To further understand the evolutionary relationships of *GATA* genes from *Arabidopsis*, rice, and tetraploid potato, a collinearity chart was constructed (**Figure 7**). It showed that 40 *StGATA* genes and 19 *AtGATA* genes were homologous gene pairs, which revealed 84 syntenic relationships, and 23 *StGATA* genes and 5 *OsGATA* genes were homologous gene pairs with 27 syntenic relationships. These results indicates that the GATA gene families of tetraploid potato and *Arabidopsis* share a close evolutionary relationship, and these genes may have similar function.

### 3.6 Expression patterns of *StGATA* genes

To explore the expression of *StGATA* genes in different tissues, we used publicly available transcriptomic data of nine different tissues (apical buds, stamens, petals, sepals, expanded leaves, stems, underground stolons, roots, and fruits) in tetraploid potato to construct an expression heat map, and the tissue-specific expression pattern with the *StGATA* genes were observed (**Supplementary Figure 3**). The *StGATA13*, *StGATA35*, *StGATA85*, and *StGATA88* genes were highly expressed in most tissues. The *StGATA15* gene was not expressed in expanded leaves but had different expression levels in other eight tissues. The *StGATA5* gene was highly expressed in the apical buds, underground stolons, and roots, while lowly expressed in petals, sepals, expanded leaves, stems, and fruits, and not expressed in stamens. The *StGATA30* gene was expressed only in sepals, whereas the *StGATA28* and *StGATA49* genes were expressed only in fruits. In addition, some *StGATA* genes were not expressed in any of the nine tissues, such as *StGATA3*, *StGATA8*, and *StGATA10* genes. These results indicates that the StGATA gene family members participate in the development of multiple potato tissues.

### 3.7 Expression analysis of *StGATA* genes in differently colored potato flesh

We performed RNA-seq analysis on differently colored potato flesh tissues to explore the potential role of *StGATA* genes in anthocyanin biosynthesis. Compared with tuber flesh of Longshu 7, a total of 18 *StGATA* genes were differentially expressed in that of Zicai 3, of which nine genes *StGATA4*, *StGATA12*, *StGATA35*, *StGATA42*, *StGATA52*, *StGATA55*, *StGATA66*, *StGATA72*, and *StGATA86* were upregulated, and the remaining genes *StGATA3*, *StGATA7*, *StGATA39*, *StGATA44*, *StGATA46*, *StGATA51*, *StGATA61*, *StGATA85*, and *StGATA87* were downregulated.

**Figure 8:**
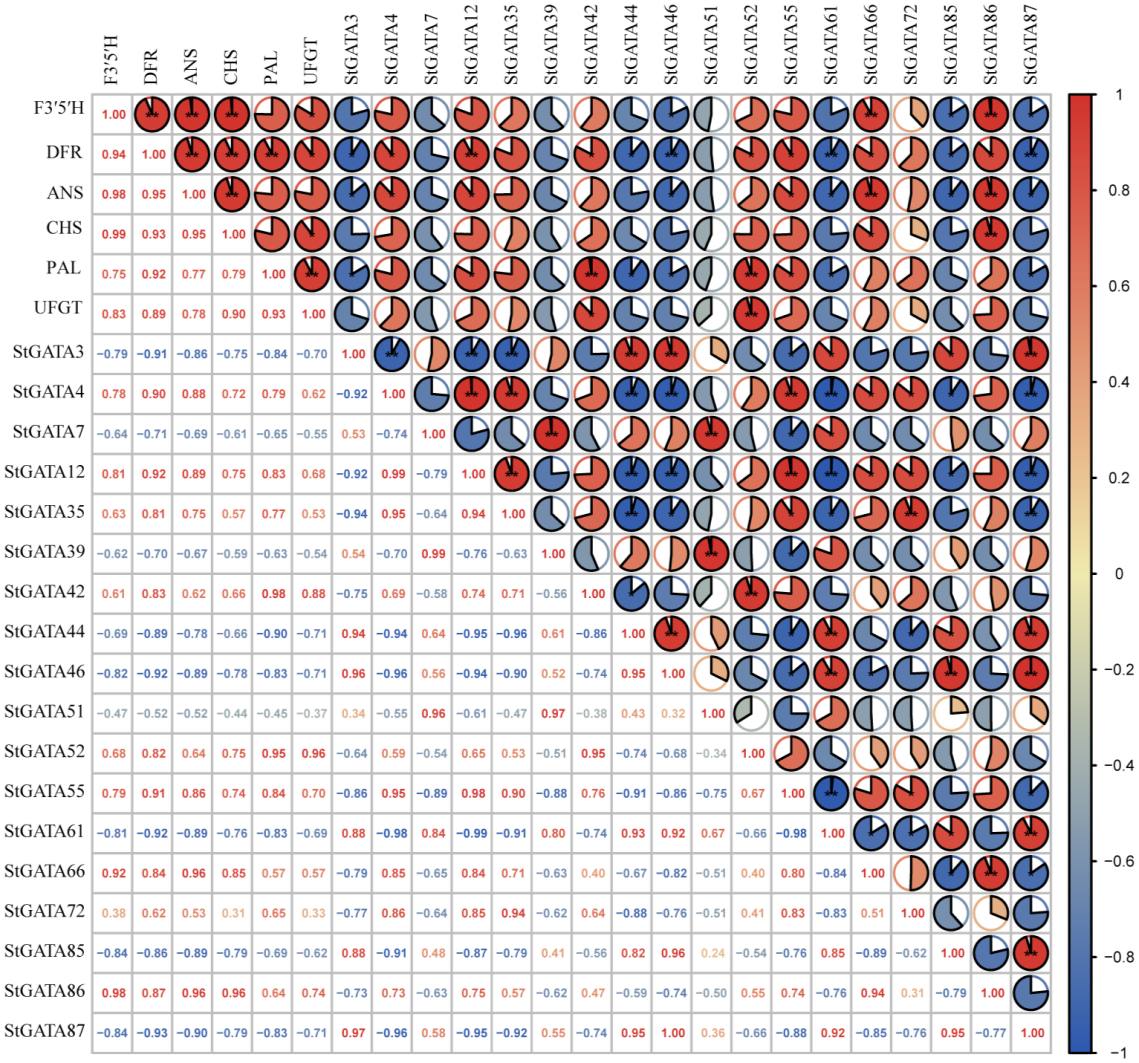
The correlation analysis of expression levels between *StGATA* genes and key genes involved in anthocyanin biosynthesis. * and ** mean significant correlation at the 0.05 and 0.01 probability levels.

We analyzed the correlation of expression levels between 18 differentially expressed *StGATA* genes in colored potato flesh and six key genes *F3’5’H (C88_C09H3G082230)*, *DFR (C88_C02H4G120570)*, *ANS (C88_C08H3G075390)*, *CHS (C88_C05H2G049530)*, *PAL (C88_C09H2G035670)* and *UFGT (C88_C09H2G045930)* for anthocyanin biosynthesis. The results showed a significant correlation between the expression levels of 14 genes (except *StGATA7*, *StGATA35*, *StGATA39*, and *StGATA51*) and key genes involved in anthocyanin biosynthesis (**Figure 8**). For example, the genes *StGATA66* and *StGATA86* were significantly positive correlated with the expression levels of *F3’5’H*, *DFR*, and *ANS* (*r* = 0.84 - 0.98), while the genes *StGATA46*, *StGATA85*, and *StGATA87* had negative and significant correlations (*r* = -0.82 - -0.93). Two genes *StGATA12* and *StGATA55* were significantly positive correlated with the expression levels of *DFR*, *ANS*, and *PAL* (*r* = 0.83 - 0.92), while *StGATA3* and *StGATA61* were significantly negative correlated with the expression levels of *DFR*, *ANS*, and *PAL* (*r* = -0.83 - -0.92). The *StGATA52* gene had a significantly positively correlation with the expression levels of key structural genes *DFR* (*r* = 0.82) and *PAL* (*r* = 0.95) in anthocyanin synthesis, and also showed a strong significantly positive correlation with the expression levels of *UFGT* (*r* = 0.96, *P* < 0.01).

**Figure 9:**
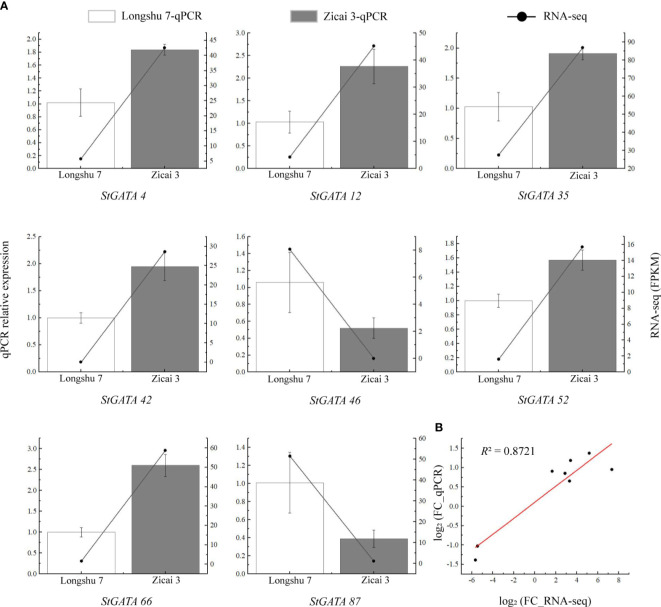
Relative expression levels of eight *StGATA* genes in **(A)** yellow (Longshu 7) and purple (Zicai 3) potato flesh. **(B)** Correlation between the qRT-PCR and RNA-seq results.

To verify the reliability of the RNA-seq data, we selected eight *StGATA* genes which were differentially expressed in purple and yellow potato flesh for qRT-PCR analysis. Although some differences occurred between the qRT-PCR and RNA-seq data, the overall trends were consistent (**Figure 9A**). We also found a strong correlation between the RNA-seq and qPCR data (*R* ^2 = ^0.8721), indicating that the transcriptome data were reliable (**Figure 9B**).

### 3.8 The tertiary structure analysis of StGATA proteins

We analyzed the protein secondary and tertiary structures of 14 *StGATA* genes related to the expression levels of key genes involved in anthocyanin synthesis. The prediction of secondary structure showed that 14 StGATA proteins were mainly composed of α-helix, β-turn, random coli and extended strand, of which α-helix and random coli were the main types, and the proportion of secondary structures were roughly similar (**Supplementary Table 5**). The prediction results of the tertiary structure indicated that all 14 StGATA proteins were monomeric proteins, with differences on overall structural similarity and moderate complexity. The results provide assistance for further understanding the tertiary structure of potato StGATA proteins (**Supplementary Figure 4**).

## 4 Discussion

GATA TFs are proteins with specific zinc finger structures that are commonly present in eukaryotes. During plant growth and development, GATA proteins help to regulate multiple biological processes, such as light signal transduction, flower development, seed germination, chlorophyll synthesis, and cytokinin-responsive (Schwechheimer et al., 2022). The GATA gene family has been reported in many different plants. For example, previous reports revealed 30 GATA family members in *Arabidopsis thaliana* (Reyes et al., 2004; Bi et al., 2005), 28 in rice (Reyes et al., 2004; Gupta et al., 2017), 28 in foxtail millet (Lai et al., 2022), 64 in soybean (Zhang et al., 2015), and 79 in wheat (Feng et al., 2022). However, primarily because commonly cultivated potato varieties are autotetraploids with highly heterozygous genomes, complicated genetic analysis and relatively scarce gene databases, the genome-wide investigation of the GATA gene family has not been performed in tetraploid potato. The previous researches on the identification of several related gene families, such as the MYB (Sun et al., 2019), bHLH (Wang et al., 2018), SBP (Kavas et al., 2017), and TCP (Wang et al., 2019) families, were mainly focused on the potato diploid genome. As the assembly of the autotetraploid potato genome was completed in 2022 (C88.v1; Bao et al., 2022), it can be used for the identification of gene family and bioinformatic analysis of the whole genome in tetraploid potato. It will help us to shed insight into the function of *GATA* genes by identifying the GATA gene family in the tetraploid potato and conducting related bioinformatic analysis. In this study, a total of 88 *GATA* genes were identified in tetraploid potato for the first time, which exceeded the number of *GATA* genes reported in diploid plants, such as *Arabidopsis thaliana*, rice, foxtail millet, and soybean. However, there was only a small difference on the number of potato *GATA* genes, compared with that of allohexaploid wheat. It is possible that autotetraploid potato has four homologous chromosomal groups, with more homologous genes than diploid species and fewer homologous genes than hexaploid species.

Based on our phylogenetic analysis of GATA proteins from different species, StGATA family members were divided into four subfamilies, of which subfamily I had the most GATA proteins, consistent with the classification of the dicotyledonous plant *Arabidopsis thaliana* (Reyes et al., 2004). The GATA gene families of tetraploid potato and *Arabidopsis* share a closer evolutionary relationship than rice. Protein domain analysis showed that the conserved domain of StGATA members in subfamily III was CX_2_CX_20_CX_2_C, whereas that in the other three subfamilies was CX_2_CX_18_CX_2_C. The similar phenomena have been observed in other dicotyledonous plants, such as *Arabidopsis* (Reyes et al., 2004), soybean (Zhang et al., 2015), and Tartary buckwheat (Yao et al., 2022). However, key differences were observed in the classifications of the GATA subfamily between monocotyledon rice and tetraploid potato. In this study, rice-specific subfamilies (V, VI, or VII) were not identified in the StGATA gene family. This result further supports the hypothesis suggested by Reyes et al. (2004) that OsGATA gene subfamilies V, VI, and VII may evolve after the divergence of monocotyledons and dicotyledons, or disappear in dicotyledons.

The conserved motifs analysis showed that the all 88 StGATA family members contained motif 1, suggesting that this motif is essential for the function of StGATA proteins. In addition, different subfamilies contained different types of conserved motifs, which led to functional diversity during evolution. In contrast, the conserved motifs of the GATA TFs in the same subfamily were generally the same, indicating that these GATA proteins may have similar function. The diversity of gene structure may play an important role in gene family evolution (Xu et al., 2012). The gene structure analysis showed that there was large variation on the numbers of introns and exons of *GATA* genes of different subfamilies. The *GATA* genes of subfamilies III and IV contained many exons and introns, of which the *StGATA8* and *StGATA16* genes of subfamily III contained 19 exons, and the *GATA* genes of subfamilies I and II contained only 2 or 3 exons. The differences on the number of the exons suggests that the StGATA gene family may generate stronger differentiation during the long-term evolution. In short, the StGATA proteins in the same subfamily have similar conserved motifs, gene structure, and phylogenetic relationships, which improves the reliability of StGATA gene-subfamily classification in our research.

*Cis*-acting elements are specific binding sites for TFs that regulate the precise initiation and efficiency of gene transcription (Tang et al., 2003; Sazegari et al., 2015). Previous report showed that GATA TFs could regulate light signal transduction by binding to light-responsive elements in the GATA promoter sequence (Luo et al., 2010). Liu et al. (2019) found that the expression of *CrGATA1* and vindoline-pathway genes was greatly induced in *Catharanthus roseus* under light conditions, because *CrGATA1* could activate the promoters of five light-responsive vindoline-pathway genes. Luo et al. (2010) found that *GATA2* was a positive regulator of photomorphogenesis, acting as a junction between light and the BR-signaling pathway, which were key signaling molecules involved in photomorphogenesis. These findings suggests that *GATA* genes may be light-regulated genes and also can respond with light regulation-related genes. In this research, light-responsive elements were present in each *StGATA* gene, suggesting that *StGATA* genes can help to regulate light response in tetraploid potato. In addition, most *StGATA* gene promoters have hormone-responsive elements and anaerobic induction-, low temperature-, and drought stress-responsive elements. Of all 88 *StGATA* genes, eight genes contained MYB-binding site elements related to the regulation of flavonoid biosynthesis. These results suggests that *StGATA* genes not only play a role in abiotic stress and hormone regulation but may also be involved in regulating flavonoid biosynthesis.

Gene duplication events (including segmental-, tandem-, and transposed-duplication events) are essential for the expansion and functional diversification of gene family during evolution (Moore and Purugganan, 2003; Cannon et al., 2004; Qiao et al., 2019). In this research, gene duplication events occurred in 87 of all 88 *StGATA* genes during evolution. All duplication events of tetroploid potato were segmental duplication, consisted with that of the wheat GATA gene family (Feng et al., 2022). Segmental duplication events were also dominant in other crops such as grape (Zhang et al., 2018), buckwheat (Yao et al., 2022), and cucumber (Zhang et al., 2021). These reports indicate that segmental duplication events may be the primary mechanism of the expansion of GATA gene family during evolution.

The collinearity analysis showed that the genomes of potato, *Arabidopsis*, and rice have many homologous gene pairs of GATA gene family. In this research, 40 potato *StGATA* genes and 19 *AtGATA* genes were identified as homologous gene pairs with 84 syntenic relationships, while 23 *StGATA* genes and five *OsGATA* genes were homologous gene pairs with 27 syntenic relationships. These homologous genes may originate from the same ancestor. Compared with syntenic relationships of *StGATA* and *OsGATA* genes, more syntenic relationships exist in *StGATA* and *AtGATA* genes, indicating that a closer genetic relationship was shared by the GATA gene families of tetraploid potato and *Arabidopsis*.

Gene expression patterns can reveal gene function to a certain extent. Shikata et al. (2004) found that the *AtGATA25* gene was involved in light signal transduction, and its overexpression promoted upward leaf extension in *Arabidopsis*. In this research, the *StGATA35*, *StGATA40* and *AtGATA25* genes were clustered together in phylogenetic tree, of which *StGATA35* and *StGATA40* genes were highly expressed in leaves (FPKM > 70), suggesting that the two genes may be involved in potato leaf elongation. Mara and Irish (2008) analyzed the expression pattern of *AtGATA21* and *AtGATA22* genes in *Arabidopsis* flower, and found that they were expressed at significantly higher levels in sepals, petals, and stamens than in other organs, which may regulate flower development. In this research, the *StGATA75* gene was clustered into a clade with *AtGATA21* and *AtGATA22* and showed differential expression in petals, stamens, and sepals, with the FPKM value from 14 to 39. It suggests that *StGATA75* gene may be involved in the regulation of flower development in tetraploid potato. De et al. (2010) found that the activation of *AtGATA23* gene promoted the expression of downstream genes related to lateral root development, which affected the lateral root elongation of *Arabidopsis*. In this research, *StGATA7* and *AtGATA23* genes were found to be orthologous genes, of which *StGATA7* gene was highly expressed in the roots of tetraploid potato (FPKM > 20), indicating that potato *StGATA7* gene may have similar function to *Arabidopsis AtGATA23* gene.

Previous findings confirmed that GATA TFs may be directly or indirectly involved in anthocyanin biosynthesis (Zhou et al., 2020; Fu et al., 2021; Wang et al., 2021; Fan et al., 2022; Zhou et al., 2022). Cho et al. (2016) revealed the regulatory mechanism of potato anthocyanin synthesis by analyzing the metabolome and transcriptome regulatory networks, and found that the transcription factor gene *GATA26 (PGSC0003DMT400066370)* played a negative regulatory role. In this research, a total of 18 *GATA* genes were screened and showed differential expression in differently colored potato flesh, of which the expression levels of 14 *StGATA* genes showed a significant correlation with that of key genes involved in anthocyanin synthesis. In addition, we found that the differentially expressed *StGATA46* and *StGATA52* genes contained an MYB-binding site element related to the regulation of flavonoid biosynthesis, respectively. It suggests that the *StGATA* genes may be directly or indirectly involved in regulating anthocyanin biosynthesis of tuber flesh in tetraploid potato, which will be further confirmed.

## 5 Conclusions

In this research, the GATA gene family in the tetraploid potato genome has been comprehensively analyzed for the first time. A total of 88 *StGATA* genes were identified, which were unevenly distributed on 44 chromosomes in tetraploid potato, and the corresponding StGATA proteins were divided into four subfamilies. A collinearity analysis indicates that segmental duplication events are the primary driving force for the expansion of the StGATA family members. The expression analysis revealed that 88 *StGATA* genes specifically expressed in different tissues. Additionally, RNA-seq analysis of potato flesh tissues with different color showed that 18 *StGATA* genes were differentially expressed, of which 14 *StGATA* genes were related to the expression levels of key genes involved in anthocyanin synthesis. The results suggestes that *GATA* genes may directly or indirectly participate in anthocyanin synthesis. In summary, this study provides rich information for better understanding the function of GATA gene family of tetraploid potato.

## Data availability statement

The datasets presented in this study can be found in online repositories. The names of the repository/repositories and accession number(s) can be found below: https://www.ncbi.nlm.nih.gov/, PRJNA1033052.

## Author contributions

XZ: Formal analysis, Investigation, Software, Visualization, Writing – original draft. RF: Investigation, Writing – original draft. ZY: Writing – review & editing, Funding acquisition, XD: Investigation, Writing – original draft. XYang: Investigation, Writing – original draft. HW: Investigation, Writing – original draft. WX: Investigation, Writing – original draft. XYu: Conceptualization, Methodology, Validation, Supervision, Writing – review & editing.

## Supplementary material

Supplementary Figure 1 Multiple sequence alignment of the GATA domains for members of the StGATA gene family.

Supplementary Figure 2 Phylogenetic tree for the GATA gene families of diploid potato and tetraploid potato.

Supplementary Figure 3 Expression levels of *StGATA* genes in different tissues. The color scale for the heat map represents FPKM values that were transformed using the following relationship: log_2_ (FPKM + 1).

Supplementary Figure 4 Analysis of tertiary structure of StGATA proteins.
