# Supplementary figures and images for: Genome-wide identification of GATA transcription factors in tetraploid potato and expression analysis in differently colored potato flesh
Source: Front Plant Sci. 2024 Mar 21;15:1330559. doi: 10.3389/fpls.2024.1330559 (PMC10991705; doi:10.3389/fpls.2024.1330559)

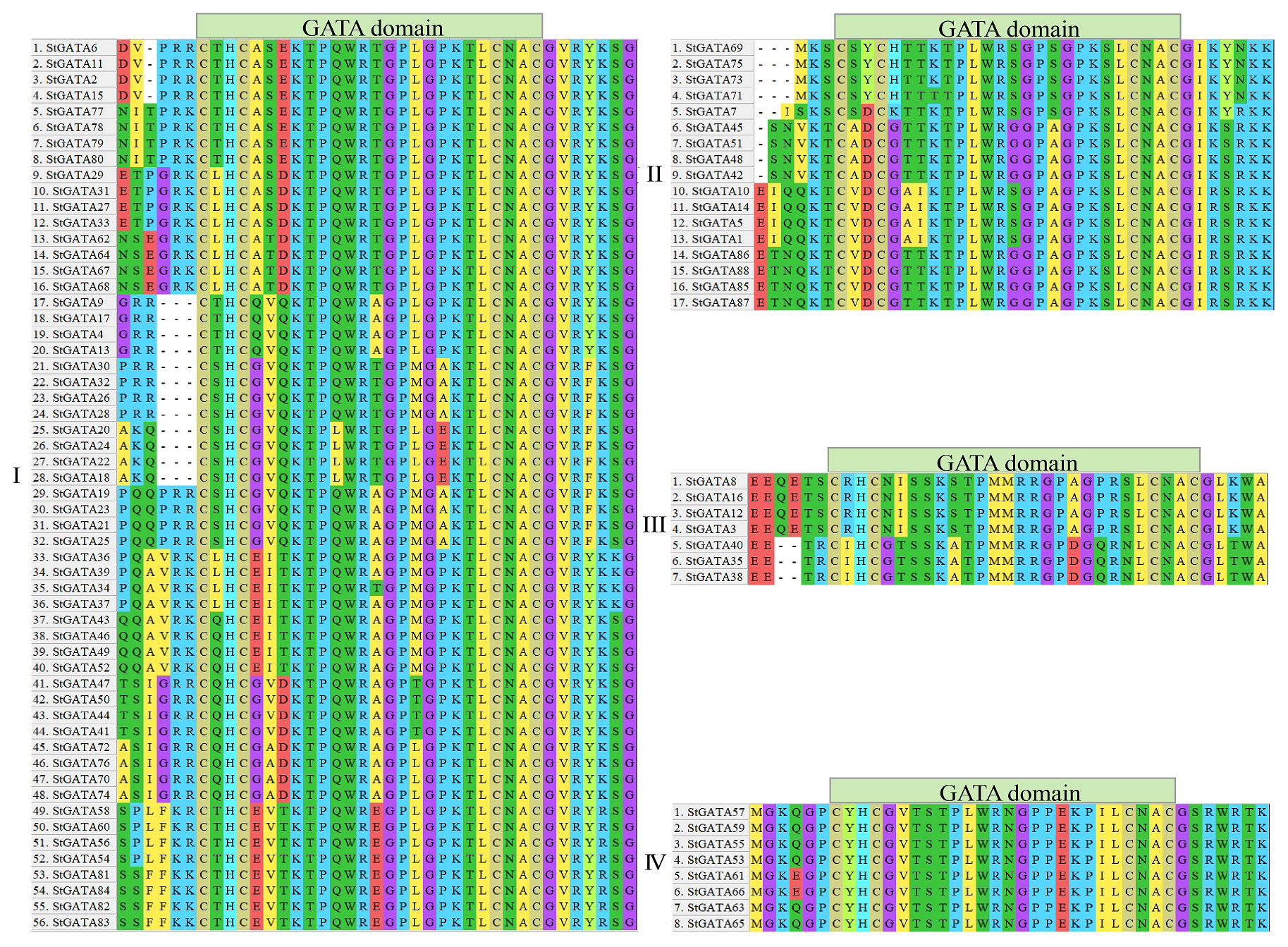

Supplement: Supplementary file 1 [file DataSheet_1.zip › supplementary material/supplementary material/Figure S1.tif]

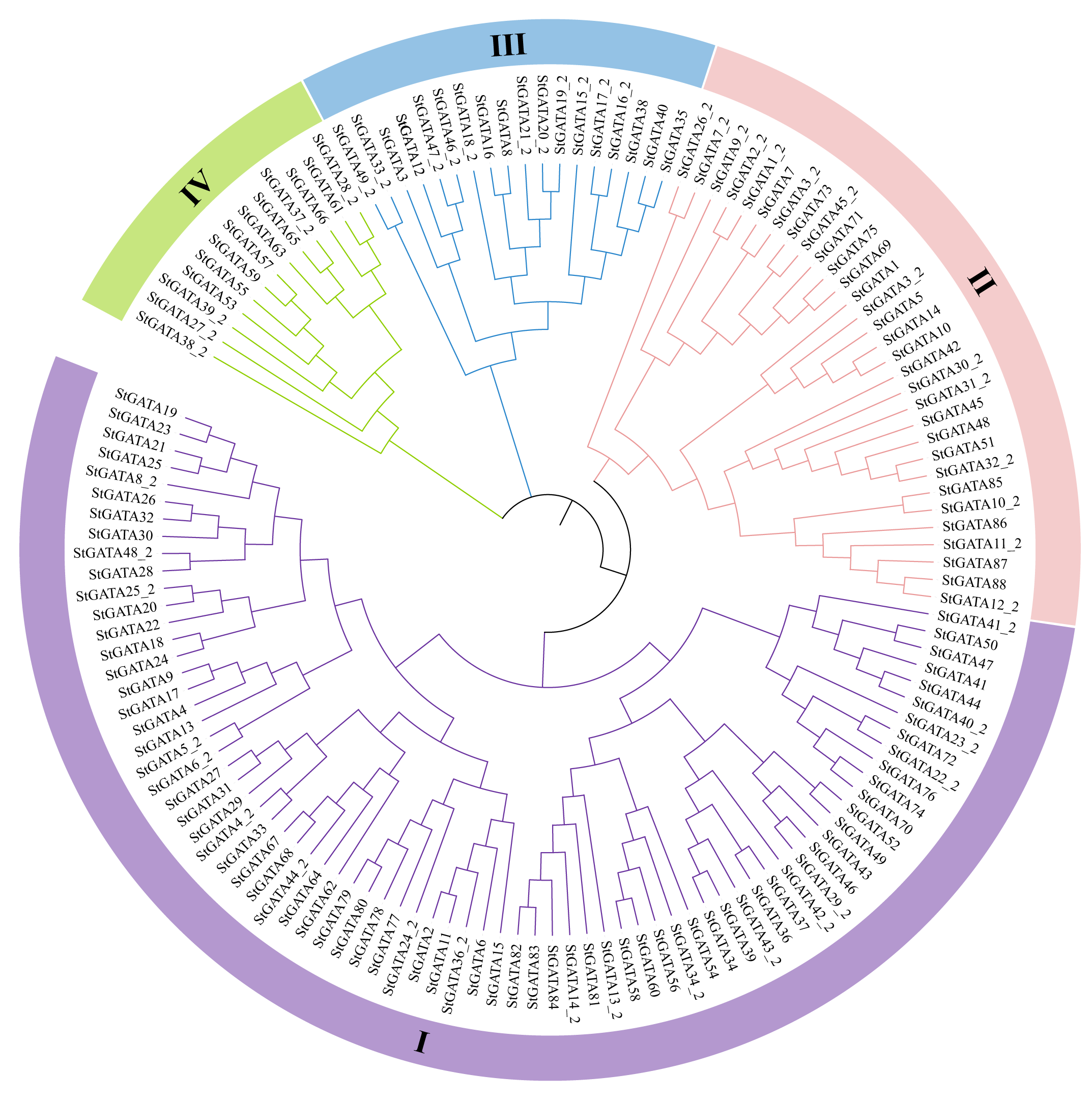

Supplement: Supplementary file 1 [file DataSheet_1.zip › supplementary material/supplementary material/Figure S2.tif]

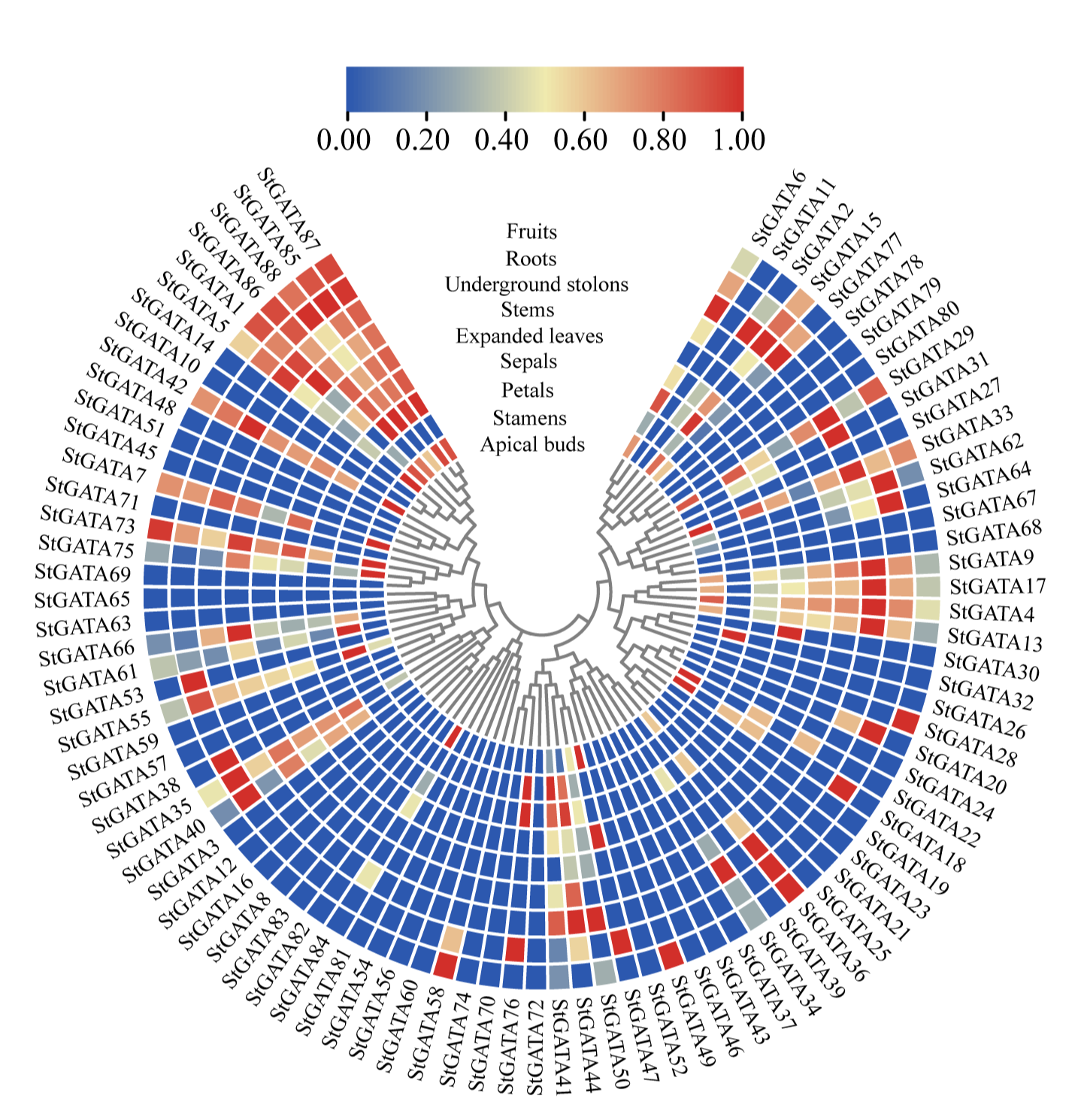

Supplement: Supplementary file 1 [file DataSheet_1.zip › supplementary material/supplementary material/Figure S3.tif]

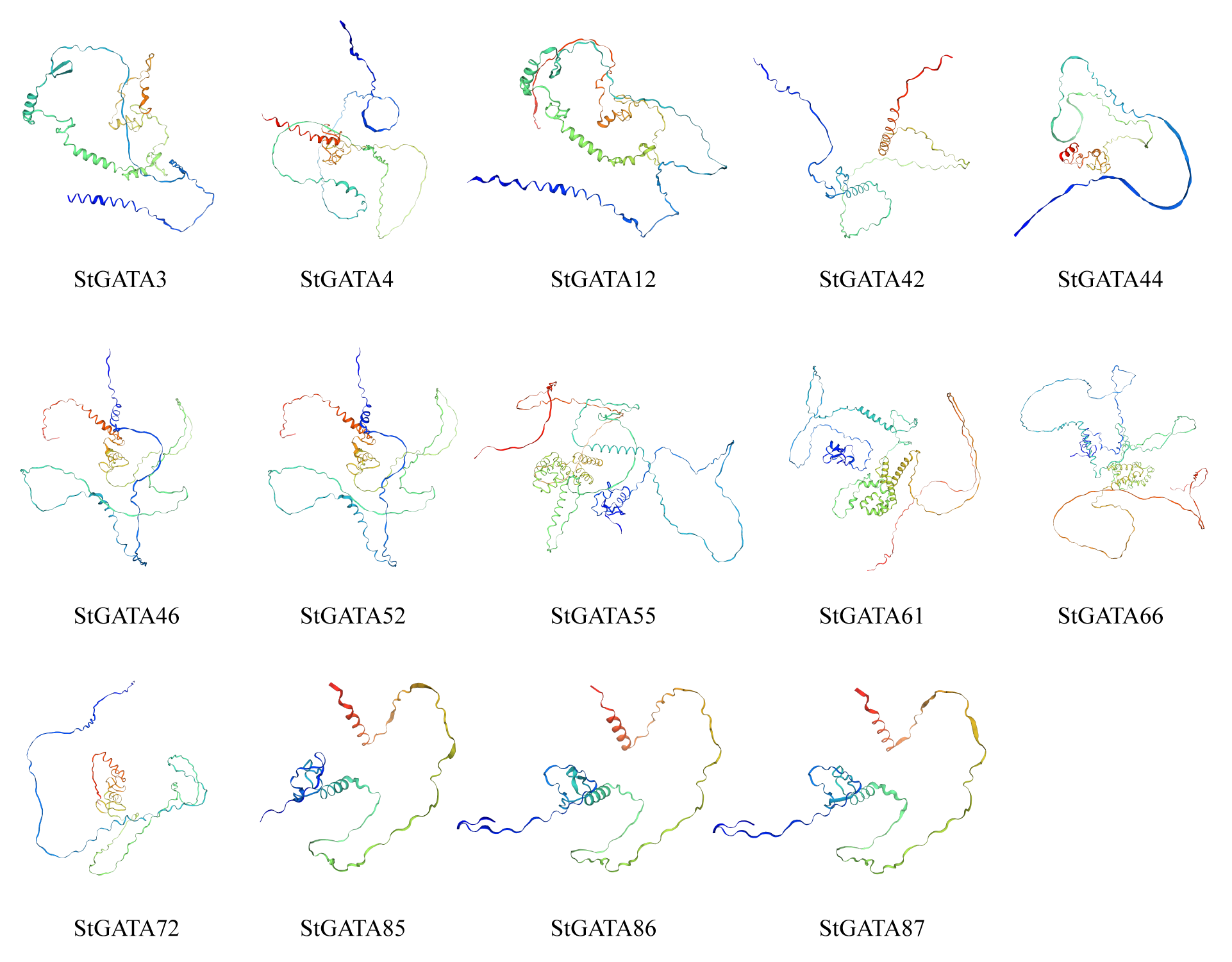

Supplement: Supplementary file 1 [file DataSheet_1.zip › supplementary material/supplementary material/Figure S4.tif]
